# Antisense oligodeoxynucleotide against human telomerase reverse transcriptase inhibits the proliferation of Eca-109 esophageal carcinoma cells

**DOI:** 10.3892/etm.2014.1897

**Published:** 2014-08-11

**Authors:** XIANG-KUI FAN, RUI-HUA YAN, BAO-JIANG LI, XIANG-MING CHEN, LIN WEI, ZHOU WANG

**Affiliations:** 1 Department of Thoracic Surgery, Shandong Provincial Hospital, Shandong University, Jinan, Shandong 250012, P.R. China; 2 Department of Tumour Surgery, Taian Central Hospital, Taian, Shandong 271000, P.R. China; 3 Coal Workers’ Sanatorium of Tanshan, Taian, Shandong 271000, P.R. China

**Keywords:** esophageal neoplasms, telomerase reverse transcriptase, antisense oligodeoxynucleotides

## Abstract

Previous studies have demonstrated that the growth of tumor cells may be inhibited by antisense oligonucleotides (ASODNs) targeted against human telomerase (hTR) or human telomerase reverse transcriptase (hTERT), resulting in antitumor activity in a wide variety of tumors. However, few studies have investigated the effect of hTERT gene-targeted ASODNs on telomerase activity and cell proliferation in human esophageal cancer. In the present study, an MTT assay was used to determine the growth inhibition rate of Eca-109 cells treated with a hTERT-targeted phosphorothioate-ASODN (PS-ASODN). An inverted microscope was used to observe the morphologic changes of the cells following treatment with 5 μM PS-ASODN for 10 days. Telomerase activity was detected using the silver staining semi-quantitative telomeric repeat amplification protocol (TRAP) assay. Following treatment with the PS-ASODN (1–5 μmol/l), the proliferation of the Eca-109 cells was inhibited. The differences in inhibition rate between the PS-ASODN and blank control groups were statistically significant (P<0.05) when the concentration of the PS-ASODN was ≥2 μmol/l, whereas no statistically significant difference was identified between the non-specific-ASODN and blank control groups. The inhibition rate increased gradually as the concentration of the PS-ASODN increased and with time, suggesting that the PS-ASODN inhibited the growth of Eca-109 cells in a concentration-dependent, time-dependent and sequence-specific manner. The growth rate of the cells incubated with the PS-ASODN was reduced compared with that of the control cells. Cells treated with the PS-ASODN became round, suspended and reduced in size. The PS-ASODN was also found to inhibit telomerase activity. The ability of the PS-ASODN to inhibit the telomerase activity and cell proliferation of the Eca-109 cell line suggests that ASODNs have the potential to be novel therapeutic agents for the treatment of esophageal cancer.

## Introduction

Telomerase is a special type of RNA nuclear protease that maintains the function of the telomere and has an important role in the immortalization of cells, as well as the genesis and progression of cancer (1,2). Telomerase is an RNA-dependent DNA polymerase that synthesizes telomeric DNA sequences, which provide tandem GT-rich repeats (TTAGGG) that compensate telomere shortening and have an important role in cellular aging and carcinogenesis. Human telomerase usually consists of three subunits: human telomerase RNA (hTR), human telomerase associated protein 1 (TEP1) and human telomerase reverse transcriptase (hTERT). The hTERT gene is the most important regulator of telomerase activity (3).

Previous studies have demonstrated that telomerase activity is absent from most normal human somatic cells, but present in >90% of tumor cells and immortalized cells (4–6). Numerous studies have shown that antisense gene therapy directed against telomerase RNA or hTERT components may effectively inhibit telomerase activity and induce apoptosis in gastric cancer, malignant gliomas, colon cancer and ovarian cancer (7–10). Previous studies have also demonstrated that the growth of tumor cells may be inhibited by antisense oligodeoxynucleotides (ASODNs) that are targeted to the hTERT gene in a wide variety of tumor types (11,12). The positive rate of telomerase expression has been found to be ≤90.48% in esophageal carcinoma tissue, whereas telomerase is not expressed in normal esophageal tissue and leiomyoma of the esophagus (13).

In the present study, a phosphorothioate antisense oligodeoxynucleotide (PS-ASODN) against hTERT was used to treat the human Eca-109 esophageal cancer cell line. The inhibitory effect and the mechanism of the PS-ASODN were investigated in the esophageal cancer cells in order to explore novel strategies for esophageal cancer gene therapy.

## Materials and methods

### Materials

Human esophageal cancer cells (Eca-109) were obtained from the Cancer Institute and Hospital (Chinese Academy of Medical Sciences, Beijing, China). RPMI-1640 medium was purchased from Gibco-BRL (Gaithersburg, MD, USA) and MTT was obtained from Sigma (St. Louis, MO, USA). Based on the sequencing of hTERT gene cDNA, an antisense sequence was designed that was complementary to the original code sequence and was 15 base pairs in length (5′-GGAGCGCGCGGCATC-3′). The original code sequence is absent from all known human genes other than hTERT. In addition, a control N-ASODN was designed (5′-ACCTGGCACCGGCGG-3′). The hTERT-targeted antisense oligodexynucleotide (PS-ASODN) and N-ASODN (all phosphorothioate modified) were provided by Shanghai Biotechnology Corporation (Shanghai, China). Liposomes (Lipofectin) were purchased from Invitrogen Life Technologies (Carlsbad, CA, USA). The semi-quantitative telomerase detection kit was obtained from the Department of Genetics of Shandong University (Jinan, China), the microplate reader model 550 was purchased from Bio-Rad (Hercules, CA, USA) and the polymerase chain reaction (PCR) thermal cycler was obtained from PerkinElmer (Waltham, MA, USA). The image scanning and analysis system was obtained from AlphaInnotech Corporation (San Leandro, CA, USA).

### Cell culture and transfection

Eca-109 cells were cultured in RPMI-1640 medium (5×10^4^ cells/ml, 37°C, 5% CO_2_ and under saturated humidity). The medium was changed every other day. Cells in the logarithmic growth phase were used for transfection.

### MTT colorimetric assay

Eca-109 cells were seeded in 96-well plates with 100 μl cell-culture medium in each well at a cell concentration of 5×10^4^/ml. Cells were cultured to the logarithmic growth phase and PS-ASODN was then added to the experimental group with final concentrations of 1, 2, 3, 4 and 5 μmol/l, while N-ASODN was added to the control group at a concentration of 5 μmol/l. RPMI-1640 cell culture medium was added to the blank control group. The cells were then cultured at 37°C, 5% CO_2_ and saturated humidity. Cells were cultured for various times (1, 5, 10 and 15 days). The cell survival rate was measured using the MTT colorimetric assay.

The absorbance (A value) was measured at a wavelength of 570 nm by a microplate reader, with the A value at a wavelength of 620 nm used as a reference, and the inhibition rate of the cells was calculated as follows: Inhibition rate (%) = A value (control group) - A value (test group)/A value (control group) - A value (blank group) ×100.

### Morphological observation

Eca-109 cells were treated with PS-ASODN (1–5 μmol/l) and N-ASODN (5 μmol/l) under culture conditions and the growth status and morphologic changes of the cells were then observed using an inverted phase-contrast microscope (Nikon Corporation, Tokyo, Japan).

### Giemsa staining

Initially, 100 μl of conventional trypsin digestion cells were extracted onto a slide glass and dried up naturally. Next, Giemsa solution was added diluted by phosphate buffer solution and stored at room temperature for 3–5 min; The rear side of the glass was rinsed and after natural drying, the glass was sealed with resin and covered with coverslips. Finally, it was observed using an optical microscope (TMS phase contrast microscope; Nikon Corporation).

### Telomeric repeat amplification protocol (TRAP)-silver staining

Cells were collected following treatment under various conditions, and the number of living cells was counted using Trypan blue staining. Approximately 1×10^4^ cells were obtained and washed using phosphate-buffered saline (PBS). The cells were centrifuged and the supernatant was discarded. CHAPS lysis buffer (10 μl) was then added and telomerase was extracted for the TRAP reaction. The procedure was as follows: the supernatant (1 μl) was added to a reaction liquid containing the forward primer (25 μl) and the reaction mixture was incubated at 30°C for 30 min in order to complete the process of telomere extension mediated by telomerase. Primers (1 μl), Taq enzyme (1 μl) and proline (20 μl) were then added. A PCR reaction was then performed under the following conditions: 94°C for 30 sec, 55°C for 30 sec and 72°C for 30 sec for a total of 30 cycles. The forward primer (TS) sequence was 5′-GGAGCGCGCGGCATC-3′, and the reverse primer (CX) sequence was 5′-ACCTGGCACCGGCGG-3′. Following PAGE gel casting, electrophoresis buffer (1X Tris-borate-EDTA) was placed in the electrophoresis tank. The TRAP amplification reaction products and loading buffer (containing bromophenol blue and xylenocyanol) were combined and electrophoresed at 200 V until the bromophenol blue reached the bottom of the gel and xylenocyanol was ~2 cm from the bottom. The gel was removed and subjected to dyeing for 10 min with 0.2% silver nitrite, fixing with alcohol for 5 min and rinsing with double-distilled water. Images were then captured for data analysis. The strength of the telomerase activity was indicated by the number of 6-bp spaced bands and the gray strength of the bands. The results from the electrophoresis were scanned and stored using SmartView (Oracle, Redwood City, CA, United States), a biological electrophoresis image analysis system. The telomerase activity of the control group was taken as 100, and the relative telomerase activity was calculated for each of the other groups. The associations of the concentration and treatment time of the PS-ASODN with the telomerase activity of the Eca-109 cells were then analyzed.

### Statistical analyses

All data were analyzed using SPSS software, version 11.0 (SPSS, Inc., Chicago, IL, USA). Variance analysis, t-tests and linear correlation were used for the analysis. P<0.05 was considered to indicate a statistically significant difference.

## Results

### Effects of PS-ASODN on Eca-109 cell growth

**Figure 1:**
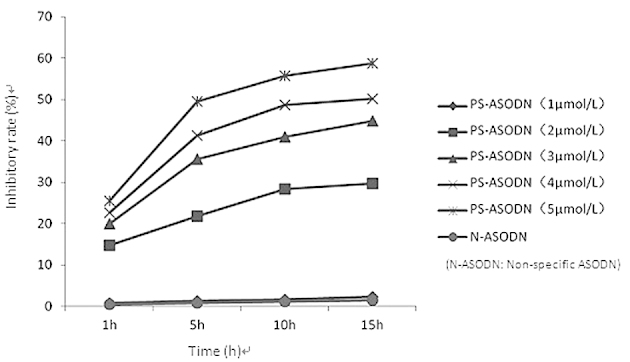
Inhibitory effect of hTERT-targeted PS-ASODN on Eca-109 cells. hTERT, human telomerase reverse transcriptase; PS-ASODN, phosphorothioate antisense oligodeoxynucleotide; N-ASODN, non-specific ASODN.

The proliferation of Eca-109 cells was found to be inhibited following treatment with PS-ASODN (1–5 μmol/l). The difference in the inhibition rate between the PS-ASODN group and the blank control group was statistically significant (P<0.05) when the concentration of PS-ASODN was ≥2 μmol/l, whereas no significant difference was observed between the N-ASODN group and blank control group (Table I, Fig. 1). The inhibition rate increased with time and as the concentration of PS-ASODN increased, which indicates that PS-ASODN inhibited the growth of Eca-109 cells in a concentration-, time- and sequence-specific manner.

### Morphological changes of the cells

**Figure 2:**
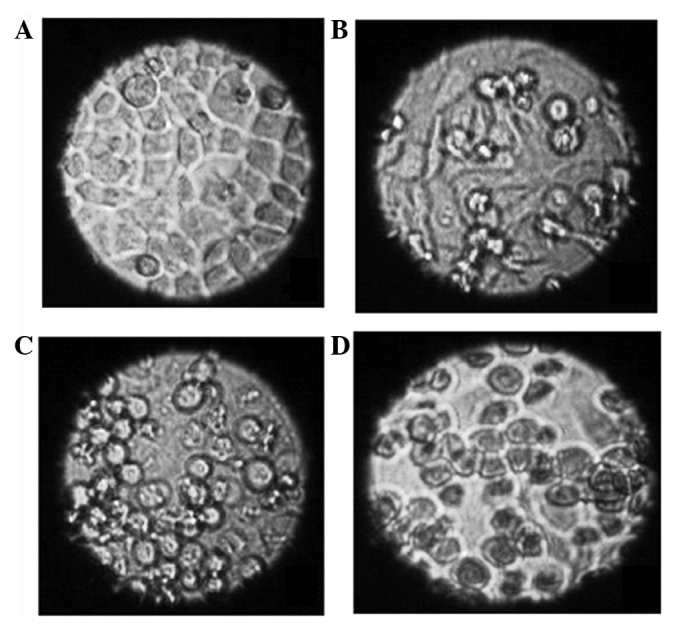
Morphological changes of Eca-109 cells. (A) Control cells; (B) cells treated with 2 μmol/l hTERT-targeted PS-ASODN for 24 h; (C) cells treated with 2 μmol/l PS-ASODN for 10 days; and (D) cells treated with 5 μmol/l PS-ASODN for 10 days. hTERT, human telomerase reverse transcriptase; PS-ASODN, phosphorothioate antisense oligodeoxynucleotide. Giemsa staining was use. Magnification, ×400.

Living cells were observed using an inverted microscope and it was found that cells treated with N-ASODN and cells in the blank control group were all epithelial and adhered to the well, the cell outlines were clear, the cells were close together and proliferation was rapid (Fig. 2A). There was no significant difference between the treatment group (2 μmol/l PS-ASODN, 24 h) and control group in morphology; however, cell growth was slower in the PS-ASODN group (Fig. 2B). After 10 days, the cells treated with PS-ASODN gradually became round and began to float in the medium and the number of floating cells gradually increased with time. Furthermore, certain cells were reduced in size, chromatin condensation was observed, the density increased and an increased number of granules was observed in the cells (Fig. 2C). These morphological changes were more marked when the concentration of PS-ASODN was increased to 5 μmol/l, and as the time of treatment was extended (Fig. 2D).

### Time-dependent effect of telomerase regulation by PS-ASODN in Eca-109 cells

**Figure 3:**
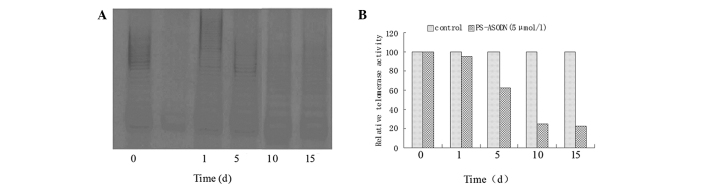
Time-effect association of telomerase in Eca-109 cells regulated by PS-ASODN. (A) Electrophoresis results showing a reduction in telomerase activity over time. (B) Quantification of the time-effect association of telomerase activity. PS-ASOD, phosphorothioate antisense oligodeoxynucleotide.

The telomerase activity decreased slightly after 24 h of treatment with 5 μmol/l PS-ASODN and the relative telomerase activity was 95 (vs. 100 at 0 h). After 5 days, the telomerase activity decreased sharply and the relative telomerase activity was 63 (37% decrease), which was significantly lower compared with the control group (P<0.05). After 10 and 15 days, the relative telomerase activity was 25 and 22, respectively (Fig. 3). By contrast, no reduction in the telomerase activity was observed in cells following treatment with 5 μmol/l N-ASODN (data not shown).

### Dose-effect association of telomerase regulation by PS-ASODN in Eca-109 cells

**Figure 4:**
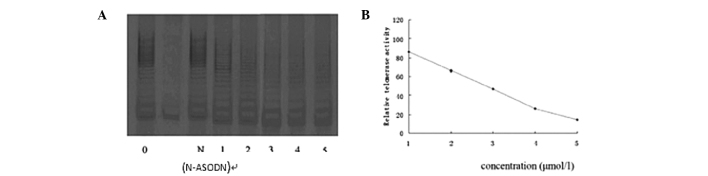
Dose-effect association of telomerase in Eca-109 cells regulated by PS-ASODN. (A) Electrophoresis showing a reduction in telomerase activity as the concentration of PS-ASODN increased. (B) The dose-effect association of telomerase activity. PS-ASOD, phosphorothioate antisense oligodeoxynucleotide; N-ASODN, non-specific ASODN.

After 10 days, the relative telomerase activity was 86, 66, 47, 26 and 14, respectively, when Eca-109 cells were treated with 1, 2, 3, 4 and 5 μmol/l PS-ASODN. The telomerase activity declined by 14, 34, 53, 74 and 86% accordingly. This indicates that as the concentration of PS-ASODN increased, the inhibitory effect on Eca-109 cells increased, which indicates that PS-ASODN acts in a concentration-dependent manner (Fig. 4).

## Discussion

High telomerase activity has been observed in malignant tumors; however, in the majority of normal tissues (with the exception of germ cells, hematopoietic cells and certain stem cells that have proliferation potential) and benign tumors, telomerase activity has been found to be low (14). The activation of telomerase is thought to be a final shared pathway for cell malignant transformation or immortalization; therefore, telomerase is considered to be an ideal target for gene therapy. Telomerase consists of 3 subunits, hTR, hTERT and TEP1. hTERT is a single copy gene that has recently been cloned and whose gene group contains a unique, highly conserved sequence. Previous studies have demonstrated that hTERT mRNA is only expressed in malignant tumors, tumor cells or certain highly potential auto-regenerated tissues (for example, the endometrium), and the expression of hTERT mRNA has been found to be correlated with telomerase activity. hTERT has been demonstrated to be specifically expressed in cancers and it has been shown to regulate the rate of telomerase activity (15). ASODNs have gained an increasing amount of attention recently due to their high specificity and targeting and low toxicity, and may potentially be used for gene therapy. ASODN gene therapy is based on the Watson-Crick principle of complementary base pairing. Artificial or biologically compounded DNA or a section of DNA is synthesized comprising a short chain of ~15–27 nucleotides that is chemically treated and complementary to the target sequence. The ASODN then targets the gene or mRNA, forming a double chain structure that inhibits transfixion or translation, thereby inhibiting the production of the protein encoded by the gene. The mechanism of ASODN treatment is as follows: i) Combination with the target DNA sequence and establishment of a tri-chain structure (U-type complementation), prohibiting the replication or transfixion of the target gene; ii) combination with the particular sequence of mRNA to form a double chain structure that prevents the ribosome from binding to the target mRNA, resulting in the inhibition of the expression of mRNA or activation of RNase H, which degrades the target mRNA by degrading the unusual double chain structure of the hetero-molecules; iii) complementing to the end coding sequence or the bottom sequence of mRNA5’ to inhibit the translation. It is essential to ensure that the ASODN is stabilized prior to its use for inhibition of the target gene. In order to do this, chemically treated oligodeoxynucleotides are introduced into the ASODN, including modifications to the phosphate backbone and pentose units (primarily at the 2-hydroxy group of ribose). Among these chemically treated derivatives, PS-ASODNs have a greatly increased resistance to degradation of the oligodeoxynucleotide by nuclease. In addition, PS-ASODNs have a good aqueous solubility, are stable and may be rapidly prepared in large quantities, and so can meet the clinical need (16,17).

A number of previous studies have used hTR as target and used ASODNs to affect the cell cultures of ovarian cancer and prostate cancer *in vitro*, and demonstrated that ASODNs inhibit telomerase activity and induce cell apoptosis (18,19). However, whether antisense hTR decreases the activity of telomerase and inhibits tumor growth in all types of cancer (for example, esophageal cancer) requires further investigation. The telomerase activity is high in the Eca-109 esophageal carcinoma cell line; however, studies concerning the effects of telomerase-targeting ASODNs in esophageal carcinoma cell lines are rare. In a previous study, we designed a PS-ASODN against hTR and applied it to Eca-109 cell cultures, and the results demonstrated that the PS-ASODN inhibited the activity of telomerase and inhibited tumor growth (20). However, hTR is widely expressed in normal tissues; therefore, adverse reactions may occur in normal cells and tissues if hTR is used as a target for antisense therapy. Previous studies have demonstrated that hTERT is the catalytic subunit of telomerase and also regulates telomerase activity. In addition, the expression of hTERT in cells and tissues has been found to be associated with telomerase activity (15,21,22). Therefore, this suggests that hTERT may be a more suitable target than hTR for antisense therapy.

Shammas *et al* (16) demonstrated that GRN163L, an antisense oligonucleotide targeting telomerase RNA, inhibited telomerase activity in bone marrow cells and induced cell death. Telomerase activity has been found to be associated with cell proliferation; therefore, the inhibition of telomerase activity may inhibit cell proliferation (23). Another study demonstrated that an antisense oligonucleotide specifically targeted against hTERT in human prostate cancer cells reduced telomerase activity, decreased proliferation and, eventually, induced cell death, indicating that hTERT is directly associated with the growth and proliferation of tumor cells (11). The interference of RNA with the expression of hTERT in cancer cells may effectively inhibit telomerase activity and strengthen the sensitivity of the cancer cells to ionizing radiation and chemotherapy (24).

In the present study, an 15-base ASODN was synthesized, which was then liposome-encapsulated and used to target hTERT in Eca-109 cells following phosphorothioate modification. The PS-ASODN was demonstrated to have an inhibitory effect on cell growth in a concentration- and time-dependent manner. However, N-ASODN had no inhibitory effect on the growth of Eca-109 cells, indicting that the inhibitory effect of PS-ASODN on cell growth was sequence specific. The results from the TRAP-silver staining assay used to detect the telomerase activity demonstrated that the telomerase activity was negatively correlated with concentration and time, and the inhibitory effect was dose- and time-dependent. Treatment with the ASODN caused the cells to decay and induced morphological changes. Using an inverted phase-contrast microscope, cells of the N-ASODN and blank groups grew well, close to the well wall and close together. The outlines of the cells were clear and spindle shapes were observed. Particles within the cytoplasm were rare and the nucleus was visible, which indicated that the cells were growing rapidly. However, in the PS-ASODN group, a number of cells were round and floating, the cells were loosely associated with vague outlines, an increasing number of particles was observed and proliferation was slow. Following Giemsa staining, the N-ASODN and blank group cells looked full, with evenly stained, clear nuclei. However, in the antisense group, the cytoplasms were condensed and rounded, and the nuclei were stained unevenly and new-moon-shaped or bulky, and contained large amounts of decayed materials.

The results from the present study demonstrate that the ASODN targeted against hTERT induced the death of esophageal neoplasm cells, and inhibited the activity of telomerase, resulting in the inhibition of cancer cell growth. The results indicate that this ASODN effectively inhibits the growth of cancer cells and may provide an experimental foundation for an anti-telomerase tumor therapy. The ASODN was designed in accordance with the target-gene sequences and base pairing; therefore, it only acted on the target gene and had no effect on other genes within the cells. There are numerous advantages to ASODNs, including high pertinence, high specificity and small size. ASODNs enter the nucleus completely and have strong anti-ribozyme activity when modified by phosphorothioates. Therefore, this technology may have broad applications.

## Figures and Tables

**Table I:** Effect of a hTERT-targeted PS-ASODN on Eca-109 cell growth (inhibition ratio, %).

Group	Concentration (μmol/l)	Time (days)			

		1	5	10	15
B	5	0.3	0.7	1.0	1.3
C (N-ASODN)	5	0.4	0.8	1.1	1.4
T1 (PS-ASODN)	1	0.8	1.2	1.6	2.2
T2 (PS-ASODN)	2	14.7^a^	21.7^a^	28.3^a^	29.6^a^
T3 (PS-ASODN)	3	20.0^a^	35.6^a^	40.9^a^	44.8^a^
T4 (PS-ASODN)	4	22.7^a^	41.3^a^	48.7^a^	50.2^a^
T5 (PS-ASODN)	5	25.4^a^	49.5^a^	55.8^a^	58.8^a^

a P<0.001, compared with group C.

B, blank control; C, control; T, treatment; hTERT, human telomerase reverse transcriptase; PS-ASOD, phosphorothioate antisense oligodeoxynucleotide; N-ASODN, non-specific ASODN.
